# Chemical and Biological Properties of Biochanin A and Its Pharmaceutical Applications

**DOI:** 10.3390/pharmaceutics15041105

**Published:** 2023-03-30

**Authors:** Zhen-Jie Feng, Wing-Fu Lai

**Affiliations:** Department of Applied Biology and Chemical Technology, Hong Kong Polytechnic University, Hong Kong SAR, China

**Keywords:** biochanin A, metabolism, extraction, bioavailability, isoflavone, chickpeas, food application

## Abstract

Biochanin A (BCA), an isoflavone derived from various plants such as chickpea, red clover and soybean, is attracting increasing attention and is considered to have applications in the development of pharmaceuticals and nutraceuticals due to its anti-inflammatory, anti-oxidant, anti-cancer and neuroprotective properties. To design optimised and targeted BCA formulations, on one hand there is a need for more in-depth studies on the biological functions of BCA. On the other hand, further studies on the chemical conformation, metabolic composition and bioavailability of BCA need to be conducted. This review highlights the various biological functions, extraction methods, metabolism, bioavailability, and application prospects of BCA. It is hoped that this review will provide a basis for understanding the mechanism, safety and toxicity of BCA and implementing the development of BCA formulations.

## 1. Introduction

Chickpeas, also known as peach beans, belong to the legume family [1]. Chickpeas have been consumed since the time of the “Fertile Crescent”, thousands of years ago [2]. They originated mainly from Southwest Asia and the Mediterranean, and are now widely distributed in 33 countries, including Australia, India and Iran [3]. Although chickpeas have now been commercialised and are popular with the public, there is still a lack of research on their breeding and processing conditions. Chickpeas have great potential for applications in food development and health promotion.

Nutritionally speaking, chickpeas are an essential source of Biochanin A (BCA) and a significant source of BCA intake for the general public. BCA is an O-methylated isoflavone and is also considered a phytoestrogen. It is present in edible plants such as chickpeas, red clover, peanuts, soybean, alfalfa, and astragalus. Among these, the highest levels of BCA are found in red clover leaves, while lower levels are found in peanut, alfalfa, and astragalus [4,5,6,7,8,9,10,11]. In recent years, with the increasing demand for high-quality diets and healthy living, BCA has become a hot research topic. Most current studies on BCA have focused on its biological functions, with a lack of research on the chemical features of BCA and the relationship of those features to biological functions. Therefore, this review fills this gap by providing a systematic description of the relationship between the chemical properties of BCA and its biological function, as well as exploring the limitations of the currently available biological studies of BCA.

## 2. Chemical Properties and Extraction of BCA

**Figure 1:**
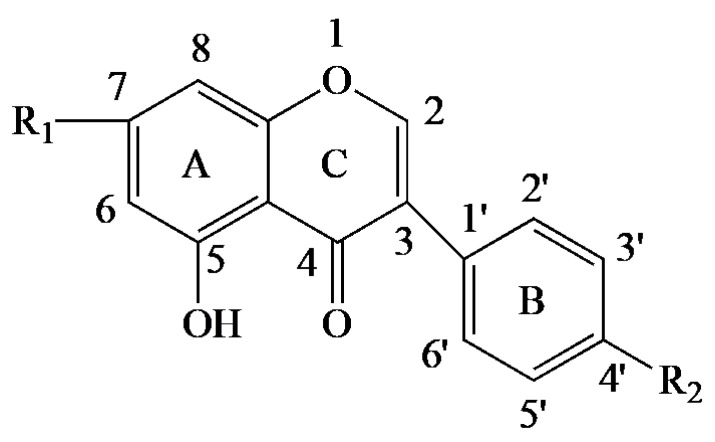
Chemical structure of BCA (R_1_ = OH, R_2_ = OCH_3_) and purnetin (R_1_ = OCH_3_, R_2_ = OH).

The chemical name of BCA is 5,7-dihydroxy-4′-methoxy-isoflavone. Its diverse biological functions are closely related to its chemical structure (Figure 1). An earlier study has shown that the anti-oxidant capacity of BCA depends on its chemical structure, and that the ketone group, as well as the position and number of attached hydroxyl groups in ring A and ring B, determine its anti-oxidant capacity [12]. The presence of an hydroxyl group at C7 in ring A, as well as the 2,3-double bond in ring C, also significantly enhance oxidative properties [12]. In addition, the methoxy group present in BCA, which exhibits electron-absorbing properties when attached to the benzene ring, can significantly increase the anti-oxidant properties of BCA [12]. It is worth noting that prunetin (Figure 1) is often overlooked as an isomer of BCA, but it also has potent biological activities. By using liquid chromatography-electrospray ionization tandem mass spectrometry (LC/ESI-MS) to identify BCA and prunetin, prunetin has been shown to improve intestinal barrier function and inflammatory response [13]. Despite the reported biological function of prunetin, in-depth studies on the mechanism of prunetin are important for the exploration of its potential applications and clinical translation.

**Figure 2:**
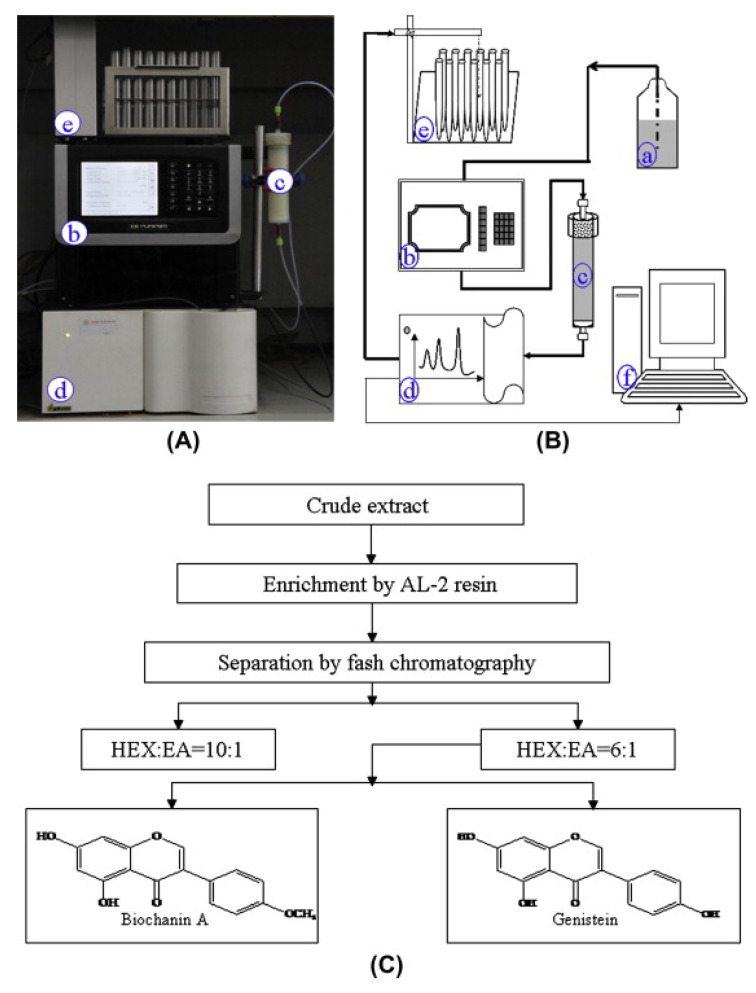
Separation and purification of BCA by flash chromatography. (**A**) A photo and (**B**) a schematic representation of the flash chromatography system: (a) mobile phase; (b) chromatography pump; (c) chromatographic column; (d) UV detector; (e) liquid reservoir; and (f) logger. (**C**) A flow chart depicting the procedures used to purify BCA. Abbreviations: HEX, hexane; EA, ethyl acetate. Reprinted with permission from Ref. [17].

A good material basis is essential for more in-depth studies on the biological functions and chemical properties of BCA. Unlike other scholars who used liquid–liquid extraction [14], ion exchange [15] or column chromatography [16] to extract the active compounds from plants, Ma and coworkers developed a simple, efficient, and low-cost method to extract BCA by using macroporous resins (Figure 2) [17]. Although 87.13% BCA yield and 95% purity were obtained using the above method, more convenient and economical extraction methods with higher yields and purity are still in dire need. This can be achieved, for example, by optimizing the selection of the mobile phase solvent and ratio, switching to a more suitable column, or using high-performance liquid chromatography (HPLC).

Apart from pristine BCA, different derivatives of BCA have potent bioactivity. Some efforts have also been devoted to modifying the chemical structure of BCA and studing its biological activity. For example, an earlier study chemically modified the structure of BCA [18]. A glycosyl group was attached to the 8-position of ring A, while a hydroxyl group (-OH) was attached to the 5-position. The modified compounds exhibited higher transferrin binding affinity [18]. By lipidating the 7 position in the BCA ring, the generated compound also significantly inhibited the proliferation of MCF-7 cells [19], suggesting that the anti-cancer biological activity of BCA could be related to the 7-position. Recently, methylation of the hydroxyl group of ring B has been found to affect the binding ability of BCA to the estrogen receptor [20]. This would affect various biological activities in vivo, such as anti-oxidant and anti-cancer properties. The in vivo form of BCA affects its bioavailability. BCA is often bound to sugars in the body to become glycosides. In the body, only glycosides are absorbed by the small intestine, bound to the oestrogen receptor, and perform further biological functions [3].

## 3. Physiological Functions of BCA

**Figure 3:**
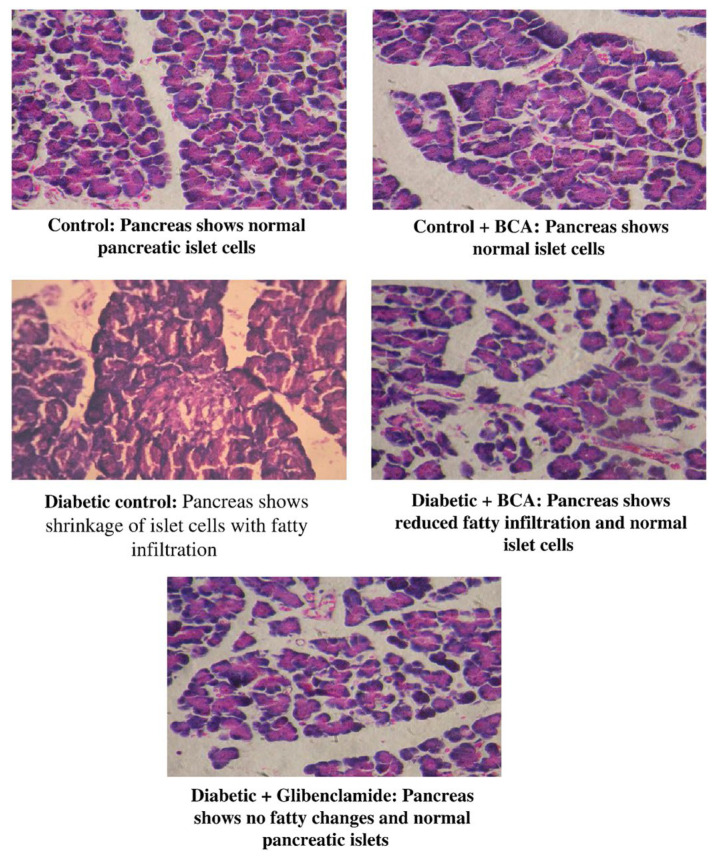
Effect of BCA on the pancreatic tissue in streptozotocin diabetic rats. Reprinted with permission from Ref. [22].

Over the years, BCA has been found to demonstrate a large variety of physiological functions. For instance, BCA has a hypoglycaemic effect for the treatment of type 2 diabetes [21]. By analysing the pancreatic tissue from BCA-treated/non-BCA-treated streptozotocin diabetic rats, it was found that the BCA-treated rats had reduced fat in normal pancreatic islet cells, indicating that BCA could act as a preventative measure against weight loss in diabetic animals (Figure 3) [22]. As a fatty acid amide hydrolase inhibitor, BCA can slow down the progression of nonalcoholic fatty liver disease (NAFLD) by regulating cholesterol metabolism [23,24]. BCA as a β-Site App-Cleaving Enzyme 1 (Bace1) inhibitor also has the potential to be developed into preventive and therapeutic agents for Alzheimer’s disease [25].

In addition, BCA is known to show anti-microbial effects. In an earlier study, BCA was found to inhibit the expression of human herpesvirus 6 antigen by inhibiting protein tyrosine kinase phosphorylation [26]. *Bifidobacterium* and *Clostridium* are part of the natural intestinal flora, and *Clostridium* may cause severe intestinal infections. In contrast, *Bifidobacterium* is a probiotic among the most critical potential bacteria in the intestine. Previously, the antibacterial activity of BCA was tested against six species of *Bifidobacterium* and eight species of *Clostridium* by in vitro assays [27]. The results showed that within the minimum inhibitory concentration (MIC) range, starting at 64 µg/mL, BCA inhibited all *Clostridium perfringens* but not *Bifidobacterium*, suggesting that BCA has a selective growth inhibitory effect in terms of antibacterial activity. However, the underlying mechanism is unclear. Many clinical isolates of *S. aureus* are resistant to a variety of antimicrobials including fluoroquinolones. By using *S. aureus* ATCC 25923 and 11 strains of fluoroquinolone (FQ)-resistant methicillin-resistant *S. aureus* (MRSA) to investigate the synergistic effect of the antimicrobial drug BCA and ciprofloxacin (CPFX) when used in combination [28], BCA and CPFX were found to have a synergistic effect against *S. aureus*, suggesting that BCA is a potential antimicrobial agent. The relationship between BCA and chlamydia has also been reported [29]. BCA was demonstrated to be an effective inhibitor of chlamydia [29]. In vitro solubility results also support that the use of BCA oral formulations could potentially improve its bioavailability in anti-chlamydial or other drug applications [29]. BCA has been shown to exhibit various health-promoting effects (Table 1), which have been linked to its anti-cancer function, anti-oxidant activity, and inflammatory function. These effects will be discussed in further detail in the following parts of this section.

### 3.1. Anti-Cancer Function

Cancer is the leading cause of death in people under the age 70 in 112 out of 183 countries worldwide [38]. According to the latest global cancer burden data published by the World Health Organization’s International Agency for Research on Cancer (IARC), there were nearly 19.3 million cancer cases and 10 million cancer deaths worldwide in 2020 [39]. BCA has been suggested to be an effective agent in treating colorectal and lung cancers. In the colon, BCA was demonstrated in in vitro experiments to be able to enhance the radiotoxicity of colon tumour cells [40]. It can also play a role in inhibiting tumour progression and immune escape by suppressing the ZEB1/PD-L1 axis [30]. In lung cancer, not only can BCA inhibit epithelial-mesenchymal transition, but it can also suppress the rate of proliferation of lung cancer cells by activating the Bcl-2 and caspase-3 pathways and by regulating the expression of cell cycle-related proteins [31,41].

**Figure 4:**
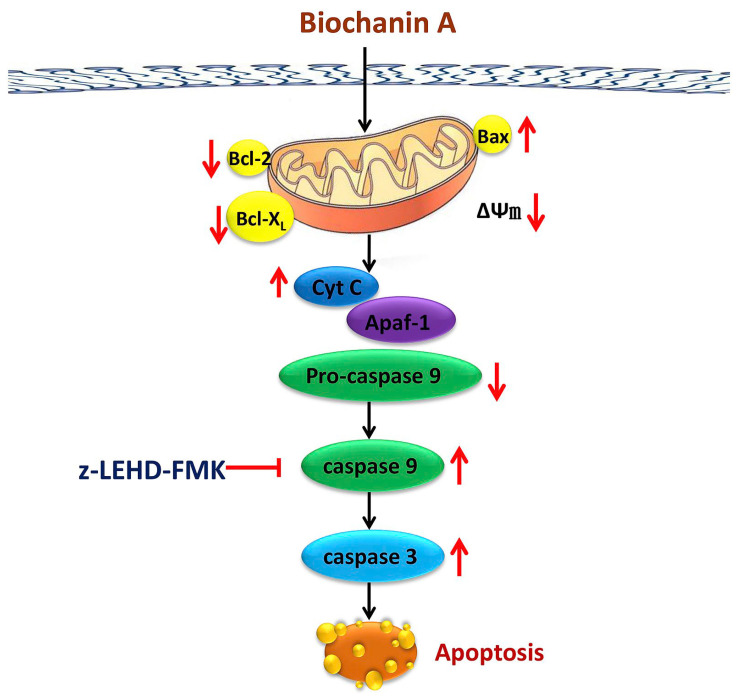
Mechanism of apoptosis of osteosarcoma cells induced by BCA. Abbreviations: Bcl-2, B-cell CLL/lymphoma 2; Bax, Bcl-2 associated X; Δψm, mitochondrial membrane potential; Cyt C, cytochrome c; Apaf-1, apoptotic protease-activating factor-1; The upwards arrows represent upregulation; The downwards arrows represent downregulation. Reprinted with permission from Ref. [7].

Apart from the cancers mentioned above, BCA has been shown to exhibit different degrees of anti-cancer properties in other types of cancers. For instance, in head and neck cancers, BCA can inhibit FaDu cell migration and proliferation by downregulating cellular signalling pathways such as p38, mitogen-activated protein kinase (MAPK), NF-κB and Akt. It can serve as a potential chemotherapeutic compound for the treatment of head and neck cancers [42]. In breast cancer, BCA is considered to be a unique natural anti-cancer agent that selectively targets cancer cells and inhibits cell viability, signalling pathways, invasive enzymes and multiple signalling pathways [32]. In myeloma, BCA binds to the CD38 protein and exerts antagonistic effect [43]. In osteosarcoma, BCA inhibited the growth of osteosarcoma cells by activating caspase 9 and caspase 3 and by increasing the ratio of Bax:Bcl-2/Bcl-X_L_ (Figure 4) [7]. In addition, as the dose of BCA increased, osteosarcoma cells were found to grow more slowly, and normal cells became less toxic [7]. This suggests that BCA has the potential to prevent and treat osteosarcoma. In glioblastoma, BCA has been shown to have sensitizing effect by modulating the AMPK/ULK1 pathway to inhibit autophagy. BCA acts as a potent sensitizer in combination with temozolomide (TMZ) to overcome the weak cellular sensitivity of TMZ alone [44]. All of these findings corroborate the potential use of BCA as an anti-cancer agent.

### 3.2. Anti-Oxidant Effects

Oxidative stress is an imbalance between the formation of oxidative free radicals in the body and the antioxidant defences of the cells. BCA displays antioxidant biological activity. It has been found to protect HepG2 cells from oxidative damage induced by tert-butyl hydroperoxide (t-BHP) [33]. In addition, in the liver of arsenic-exposed rats, an increase in lipid peroxidation responses, accompanied by depletion of catalase (CAT) and superoxide dismutase (SOD) activities, has been reported [45]; however, administration of BCA (20 mg/kg-bw/day), along with selenium (3 mg/kg-bw/day), has been shown to reverse liver and oxidative stress markers in those rats. Although low doses of BCA (10 mg/kg-bw/day) did not show any preventive effect, high doses of BCA (40 mg/kg-bw/day) played a role in preventing hepatotoxic events [45]. All these demonstrate the potential use of BCA in combating oxidative stress-mediated pathological events.

Moreover, exogenous toxins such as bisphenol A (BPA) can easily trigger oxidative damage leading to neurological problems [46]. It has been shown that BPA reduces glutathione levels, while the addition of BCA increases the metabolism of glutathione and facilitates the scavenging of oxygen free radicals, thus reducing oxidative damage [46]. In addition, prolonged exposure to BPA triggered neuroinflammation and thus recruitment of immune cells; however, co-treatment with BCA resulted in a reduction in macrophage recruitment [46]. Along with the fact that, in SH-SY5Y cells, BCA has been found to activate the Nrf2/ARE pathway to inhibit isoflurane-induced oxidative stress and prevent neurotoxicity [34]. This makes BCA a promising candidate for further exploitation to tackle neurodegenerative diseases caused by oxidative stress in the future.

### 3.3. Anti-Inflammatory Function

Inflammation is considered a defensive response of body tissues to stimuli from different damage factors, and is an essential self-regulatory process for maintaining the homeostasis of the body’s internal environment. In rat midbrain neuron-glia cultures, BCA has been reported to be effective not only in reducing lipopolysaccharide (LPS)-induced reductions in dopamine uptake and the number of dopaminergic neurons, but also in inhibiting lipopolysaccharide-induced microglial activation of tumour necrosis factor, nitric oxide and superoxide production by microglia [35]. It can also effectively alleviate gingival inflammation, a common oral disease, in rats by inhibiting TNF-ɑ, IL-1β, ROS and elevating OCN a and Nrf2 levels [36].

**Figure 5:**
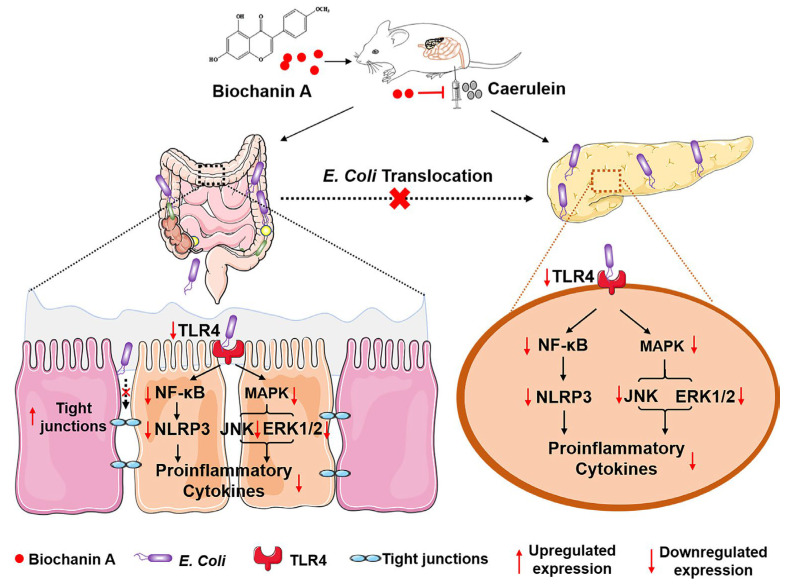
Mechanisms of BCA to prevent AP and intestinal injury. Reprinted with permission from Ref. [37].

With a rapid increase in the incidence of idiopathic pulmonary fibrosis (IPF), there is an urgent need for new drugs to replace pirfenidone, which has many adverse side effects [47]. BCA has been found to significantly reduce the expression of TGF-β-regulated fibrotic genes and to reduce the expression of inflammatory markers [48]. By comparing the therapeutic efficacy of BCA with that of pirfenidone, BCA was more effective in ameliorating pulmonary fibrosis [48]. This provides a new direction for the treatment of IPF. Finally, the incidence of acute pancreatitis (AP) is on the rise, and the huge cost to the healthcare system has attracted our attention [49]. Patients with AP have high serum pancreatic enzymes that can lead to multi-organ dysfunction, and many AP patients require repeated visits to the clinic, severely affecting their quality of life [49]. In a mouse model, BCA has been reported to reduce the migration of pathogenic *Escherichia coli* (*E. coli*) to the pancreas and to inhibit TLR4-MARK/NF-κB signalling and activation of the NLRP3 inflammasome [37], thereby preventing AP and intestinal damage (Figure 5). BCA could be a potential drug for the treatment and prevention of AP.

## 4. Metabolism and Strategies to Enhance the Bioavailability of BCA

The elucidation of processes involved in the metabolism of BCA can help us to better understand the biological functions and mechanisms of action of BCA. Metabolism can be divided into phase I and phase II. Phase I metabolism, also known as biotransformation, is a process in which either a new functional group is introduced to a molecule, or a small existing group is removed. Common types of phase I metabolism reactions include oxidation, reduction and hydrolysis. Phase II metabolism involves the binding of some endogenous components to an agent or phase I metabolite to form a conjugate that can be effectively excreted from the body. Representative types of phase II metabolism include glucuronidation and acetylation. The metabolism of BCA has previously been studied using gas chromatography–mass spectrometry (GC-MS) [50]. Seven BCA metabolites have been identified from red clover in human urine [50]. Two BCA metabolites have also been found in human liver microsomes [51]. Recently, by using ultrahigh-performance liquid chromatography coupled with quadrupole time-of-flight mass spectrometry (UHPLC-Q-TOF-MS/MS) [52], a total of 43 metabolites in rats, 22 metabolites in liver microsomes, and 18 metabolites in intestinal flora were elucidated [52]. Glucuronidation, sulphonation and methylation were found to be the primary forms of reaction for BCA phase II metabolism. By examining the levels of BCA in several samples in vivo, BCA was found to be the least abundant in blood (0.96%), followed by bile (1.38%) and urine (45.10%), further suggesting that BCA undergoes a wide range of metabolic patterns in different organs in vivo [52]. In addition, the levels of BCA metabolites in different organs were also assessed, and the lowest levels of BCA metabolites were found in the intestinal flora.

**Figure 6:**
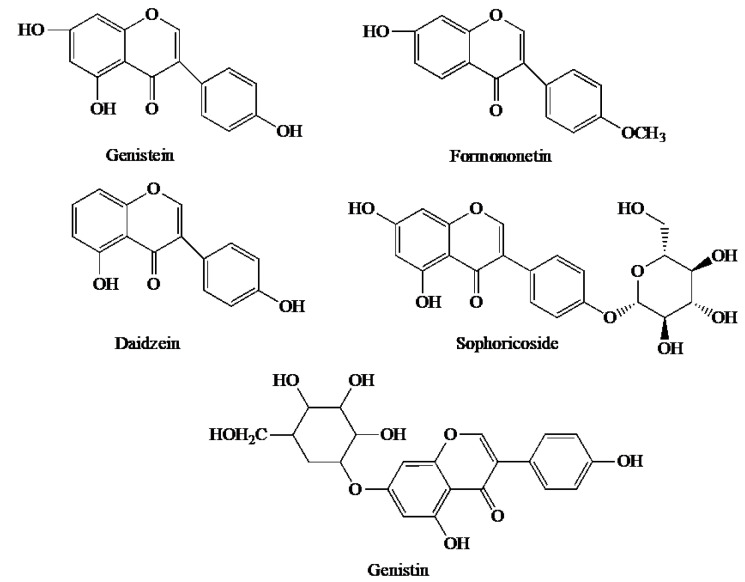
Chemical structures of 5 important BCA metabolites.

Formononetin, genistein, daidzein, sophoricoside and genistin are thought to be five biologically important BCA metabolites (Figure 6) [52]. They are all isoflavones. Genistein can be detected directly in the blood and undergo various further metabolic reactions. Genistein is well known for its anti-tumour, anti-inflammatory and anti-oxidant activities [29]. Formononetin has been reported to be a neuroprotective agent not only for the treatment of Parkinson’s disease but also for its hypolipidemic, anti-osteoporotic and anti-cancer effects [53]. Daidzein has been reported to inhibit the invasion of MDA-MB-231 breast cancer cells by reducing matrix metalloproteinase (MMP)-2 activity [54]. It has also been reported to have antithrombotic, antiallergic, anti-oxidant and antidiabetic effects [54]. Sophoricoside has been found to be able to regulate adipogenesis and glucose consumption, whereas genistin has been shown to have anti-lipidemic effects [55]. Given the diversity and complexity of BCA metabolites, elucidation of BCA metabolism would be one of the research directions that are worth paying attention to. A good understanding of the metabolism of BCA can provide the basis for future clinical applications of BCA.

In fact, BCA is characterised by poor oral absorption and low bioavailability. This limits the development and use of BCA in food and pharmaceutical applications [56,57,58]. Since the turn of the last century, the possibility of manipulating properties of bioactive agents have been aided by biotechnological [59,60,61,62,63] and materials innovations [64,65,66,67,68,69]. This sheds light on strategies to enhance the bioavailability of BCA. For example, solid dispersions of BCA were prepared using Solutol HS15 and HPMC 2910 [70]. The dissolution rate and drug release of BCA were significantly improved, and its bioavailability was increased by 8–60 times. By encapsulating BCA in micelles containing Pluronic F127 and Plasdone S360, the oral bioavailability of BCA was increased by 2.16 times [71], revealing that nanomicelles can be used as a delivery system for BCA. In addition, ionic gelation was used to micronise BCA [72]. Upon being coated with ethylcellulose, BCA can be delivered directly to the intestine by bypassing gastric degradation and first-pass effects, resulting in an increase in oral bioavailability [72]. Recently, electrospun polylactide (PLA) fibres were used to deliver BCA in a sustained-release manner [73]. Cyclodextrins have also been adopted to form inclusion complexes with BCA, so as to improve the solubility and bioavailability of BCA [74]. Apart from the above-mentioned systems and formulations, studies have shown that co-administration of various isoflavones such as BCA, genistein, daidzein, coumestrol and zearalenone can increase bioavailability by up to 28% [75]. This provides an opportunity to conduct studies on improving the bioavailability of BCA in the body.

## 5. Opportunities and Challenges for Applications of BCA and Its Sources

Over the years, various studies have been conducted evaluating the safety profile of BCA. For example, in a previous study, three different dosages of BCA (3 mg/kg body weight; 10 mg/kg body weight; 30 mg/kg body weight) were orally administered to rats for 28 days [76]. After that, general toxicity parameters were studied by examining clinical signs, body weight, and organ weight [76]. Comparison between the experimental and control groups showed no clinical toxicity at day 28 and no changes in body weight or organ weights (liver, kidney, heart, lung) [76]. Recently, a novel BCA-chromium (III) complex was generated to treat diabetes [77]. The complex was found to show no effect on serum parameters, organs or anti-oxidant capacity in mice [77]. All this suggests that BCA is potentially safe for biomedical use. Despite this, all of the above studies were limited to mouse models. To date, the activity of BCA has yet to be thoroughly evaluated in humans [57]. This means that although BCA can have some benefits as a food supplement, the potential risks of BCA in humans need to be further investigated. In fact, it is important to know the toxicity of BCA before applying it clinically for disease prevention and treatment. A more in-depth assessment of BCA toxicity and a better understanding of the toxicological mechanisms of BCA in human models are direly needed for the future development of BCA-based pharmaceutical products.

Apart from its possible pharmaceutical applications, BCA has been widely exploited as a functional ingredient in nutritional supplements. Companies have commercialised BCA for use in nutritional supplements specifically to alleviate the symptoms that occur in women after menopause [57]. It is worth noting that no BCA-only products are currently available on the market. Instead, BCA is only used as one of the ingredients in products. Furthermore, nutritional supplements are generally not subject to rigorous standardized and quality control measures. For this, the quality of dietary supplements on the market that contain BCA varies, and they may be adulterated [78]. Manufacturers may also not be able to properly guide consumers on the intake of nutritional supplements due to the lack of standardised rules on the amount to be added. Regulating the market for nutraceuticals containing BCA will, therefore, be the future trend and the way forward.

Apart from taking BCA per se, oral administration of the botanical sources of BCA (particularly chickpeas which serve as an important dietary source of BCA among the general public) may lead to health benefits owing to the effects of the BCA inside. Besides the most common method of consuming chickpeas, i.e., ground into a bean puree and packaged in a can, chickpeas can be added as functional ingredients to foods [79]. For instance, chickpeas can be ground into flour for making biscuits, bread, and pasta. A study showed that, compared to those produced using conventional wheat flour, bread and biscuits produced using chickpea-containing flour demonstrated lower amounts of acrylamide during the baking process [80]. By blending extruded chickpea flour with nixtamalized maize flour from protein maize, infant foods with good protein digestibility have been successfully generated [81]. Despite this application potential, difficulties in cultivating chickpeas have hindered wide applications of chickpeas in functional food development. Over the last 20 years, chickpea yields have remained at 700–800 kg/ha, with a very slow growth rate of 1.3% per year partly due to the infestation of chickpeas [82]. In addition, when too much chickpea flour is used in bread, degradation of starch granules and increased enzyme activity result, making the food products less appetising to consumers [83]. In the future, generating a good variety of genetically modified chickpeas through the collaboration of multiple stakeholders may be a possible direction to improve chickpea yields and to streamline the development of chickpea-based functional food products [82]. Meanwhile, effective improvement of chickpea stability and extensibility will not only provide the maximum nutritional value of chickpeas but also improve the texture, taste and colour of chickpea-based food products.

## 6. Concluding Remarks and Outlooks

Over the years, studies have shown that BCA has anti-inflammatory [36], anti-oxidant [45], and anti-cancer [32] properties, and serve various biological functions (including inhibition of pathogenic microorganisms [26], neuroprotection [34], and treatment of arthritis [84]). However, there are still areas to be further investigated. Firstly, in terms of inhibition of pathogenic microorganisms, BCA alone has shown many negative results in antimicrobial therapy [28]. The reason may be that BCA relies mainly on synergistic effects with other drugs to work. At the same time, this illustrates the complexity of the role of BCA in the organism. The specific anti-disease mechanism of BCA must, therefore, be elucidated. Secondly, in terms of its anti-cancer properties, considering that cancer is a heterogeneous oncological disease, we believe that there is variability in the molecular mechanisms of BCA in different cancers and their subtypes [30,31,43]. The efficiency of BCA in combating different cancers should be examined empirically. Thirdly, in terms of its anti-inflammation properties, existing studies are limited to cellular and animal models [35,36]. No studies on the anti-inflammatory effects of BCA on humans have been conducted. In addition, considering the complexity of the inflammatory response in a body, studies on whether BCA has any pro-inflammatory effects in humans and the relationship between BCA and different types of inflammation are needed. Apart from this, few studies have been conducted to extensively elucidate processes involved in BCA metabolism and there are discrepancies between reports on BCA metabolites [50,51,52,85]. Given the diversity and complexity of BCA metabolites, further studies on BCA metabolism are necessary. Moreover, biological function is closely related to the chemical structure. However, few studies have examined the biological activity of BCA after chemical modification [18,19,20].

To design and optimize BCA-based formulations, more in-depth studies on the structure–activity relationship of BCA are direly needed. In terms of bioavailability, the low bioavailability of BCA has limited its development and application in the food and pharmaceutical sectors. Developing strategies to effectively enhance the bioavailability of BCA is an important area in future research. Meanwhile, the effectiveness of BCA has not been adequately evaluated in humans. Further research is needed on the SPL and potential risks of BCA to humans. Last but not least, chickpeas are an important source of BCA. However, partly due to the limitations in chickpea cultivation and processing [83], they are not fully exploited for food and pharmaceutical applications. Developing a variety of genetically modified chickpeas to improve the yield and in-depth research into processing techniques could help streamline chickpea product development. We believe that making the most of the BCA and its natural sources will be a significant commercial opportunity.

## Figures and Tables

**Table 1:** The properties of BCA and their mechanisms of action.

Property	Mechanism	Ref.
Anti-cancer effect	Inhibit tumour progression by suppressing the ZEB1/PD-L1 axis	[30]
	Activate the Bcl-2 and caspase-3 pathways.	[31]
	Inhibit the expression of invasive enzymes and modulate multiple signal-ling pathways in the Her-2-positive breast cell line SK-BR-3.	[32]
	Inhibit the growth of osteosarcoma cells by activating caspase 9 and caspase 3 and by increasing the ratio of Bax:Bcl-2/Bcl-X_L_.	[7]
Anti-oxidant effect	Protect HepG2 cells from tert-butyl hydroperoxide (t-BHP)-induced downstream cytoprotective enzymes, including NQO1 and HO-1.	[33]
	Activate the Nrf2/ARE pathway, leading to a decrease in the levels of SOD and CAT, and an increase in MDA, Nrf2, HO-1 and NQO1 levels.	[34]
	Reduce lipopolysaccharide (LPS)-induced dopamine and inhibit LPS-induced microglia-activated production of necrotizing factor, nitric oxide and superoxide.	[35]
Anti-inflammatory effect	Inhibit TNF-ɑ, IL-1β, ROS and elevate OCN a and Nrf2 levels.	[36]
	Inhibit TLR4-MARK/NF-κB signaling and NLRP3 inflammasome activation.	[37]
Hypoglycemic effect	Increase SIRT1 expression in the pancreatic tissue.	[21]

## Data Availability

No new data were created or analysed in this article. Data sharing is not applicable.
